# Alcohol consumption, body mass index and breast cancer risk by hormone receptor status: Women’ Lifestyle and Health Study

**DOI:** 10.1186/s12885-015-1896-3

**Published:** 2015-11-09

**Authors:** Aesun Shin, Sven Sandin, Marie Lof, Karen L. Margolis, Kyeezu Kim, Elisabeth Couto, Hans Olov Adami, Elisabete Weiderpass

**Affiliations:** 1 Department of Preventive Medicine, Seoul National University College of Medicine, Seoul, South Korea; 2 Department of Medical Epidemiology and Biostatistics, Karolinska Institutet, PO Box 281, 171 77 Stockholm, Sweden; 3 Department of Biosciences and Nutrition, Karolinska Institutet, Stockholm, Sweden; 4 HealthPartners Institute for Education and Research, Minneapolis, USA; 5 Norwegian Knowledge Centre for the Health Services, Health Economic and Drug Unit, Oslo, Norway; 6 Department of Epidemiology, Harvard School of Public Health, Boston, USA; 7 Department of Research, Cancer Registry of Norway, Institute of Population-Based Cancer Research, Oslo, Norway; 8 Department of Community Medicine, Faculty of Health Sciences, University of Tromsø, The Arctic University of Norway, Tromsø, Norway; 9 Genetic Epidemiology Group, Folkhälsan Research Center, Helsinki, Finland

**Keywords:** Breast cancer, Alcohol, Body mass index, Hormone receptor

## Abstract

**Background:**

We aimed to estimate the effect of alcohol consumption on breast cancer risk and to test whether overweight and obesity modifies this association.

**Methods:**

We included in the analysis 45,233 women enrolled in the Swedish Women’s Lifestyle and Health study between 1991 and 1992. Participants were followed for occurrence of breast cancer and death until December 2009. Poisson regression models were used, and analyses were done for overall breast cancer and for estrogen receptor positive or negative (ER+, ER-) and progesterone receptor positive and negative (PR+, PR-) tumors separately.

**Results:**

A total of 1,385 breast cancer cases were ascertained during the follow-up period. Overall, we found no statistically significant association between alcohol intake and breast cancer risk after adjustment for confounding, with an estimated relative risk (RR) of 1.01 (95 % CI: 0.98–1.04) for an increment in alcohol consumption of 5 g/day. A statistically significant elevated breast cancer risk associated with higher alcohol consumption was found only among women with BMI ≤25 (RR 1.03, 95 % CI 1.0–1.05 per 5 g/day increase).

**Conclusion:**

An increase in breast cancer risk with higher alcohol consumption was found for breast cancers in women with a BMI ≤25 kg/m^2^.

## Background

The International Agency for Research on Cancer (IARC) has classified alcohol as a human carcinogen [1] which increases risk of breast cancer both before and after menopause [2]. Alcohol consumption has been estimated to account for 5 % of breast cancer incidence in the European Prospective Investigation into Cancer and Nutrition (EPIC) study [3]. Population attributable fraction of alcohol on breast cancer mortality has been estimated to be 6.4 % in the United Kingdom,[4] 9.4 % in France,[5] and 6 % in the United States [6].

Several mechanisms have been proposed to explain this association [2] increasing the probability that alcohol might play a causal role in breast cancer etiology. According to one theory, alcohol increases circulating estrogen levels through interaction with estrogen metabolism [7]. The same mechanism, augmented by decreased levels of sex hormone binding globulin (SHBG) leading to more available estrogen, is also proposed to explain why obesity increases risk of postmenopausal breast cancer [8]. Before menopause, obese women experience a lower risk for breast cancer than lean women; however, the opposite is observed after menopause [2, 9]. The inverse association between obesity and risk of premenopausal breast cancer is less well understood mechanistically.

We hypothesized that if alcohol and obesity compete via a similar mechanism to increase risk of postmenopausal breast cancer, then these two exposures may not be additive. We further hypothesized that the possible association between these exposures and breast cancer may pertain chiefly or exclusively to hormone receptor positive cancers. We tested our hypotheses in a large prospective study in Sweden.

## Methods

### Study population

The study cohort comprised participants of the Swedish Women’s Lifestyle and Health (WLH) study (http://ki.se/en/meb/womens-lifestyle-and-health). Details of the study design have been described elsewhere [10, 11]. The study enrolled women age 30–49 who resided in the Uppsala Health Care Region. Among 49,259 women who answered the baseline questionnaire in 1991 and 1992 we excluded 374 for the following reasons: death (*n* = 68), emigration without re-immigration (*n* = 67), previous breast cancer diagnosis (*n* = 273), and uninterpretable answers to questionnaire (*n* = 2). We further excluded 3,652 women with any missing value on selected key covariates (age, birth year, weight, height, education, family history of breast cancer, alcohol consumption, smoking habit, age at menarche, menopausal status, age at menopause, parity, age at the first birth, breast feeding duration, and oral contraceptive use). The final analytic cohort consisted of 45,233 women. Participants were asked to report the number of glasses of beer, wine, and spirits that she currently drank per week, per month, or per year on the baseline questionnaire. On the follow-up questionnaire, consumption frequency and the amount of consumption on one occasion for low alcohol beer, beer, white wine, red wine, dessert wine, and spirits were asked. Body mass index was calculated by using the present height and weight reported on the baseline questionnaire.

Complete follow-up was achieved through linkage to the nationwide health registries in Sweden using the unique national registration number assigned to each individual. Overall breast cancer incidence was obtained from the national register, and estrogen receptor (ER) and progesterone receptor (PR) status of breast tumor was obtained from the regional register. Incident invasive breast cancer (ICD7 170) was ascertained from the Swedish cancer register from 1st September 1992 when the regional breast cancer register in Uppsala was set up, up to 31st December 2009. The Swedish Data Inspection Board and the regional Ethical Committee, Uppsala University, Uppsala, Sweden and the Ethical Committee of the Karolinska Institutet in Stockholm, Sweden approved the study protocol. All participants signed an informed consent form.

### Statistical analysis

We analyzed breast cancer risk associated with alcohol consumption using Poisson regression. This was done with alcohol intake modeled without any predefined shape, with step-function using alcohol categories and also as linear continuous form. To evaluate the functional form between alcohol intake and breast cancer incidence, we estimated the relative risk (RR) using splines, i.e. log (cancer rate) = h (alcohol, gram/day) where h () is an arbitrarily shaped curve. We fitted all models adjusting for age and for other possible confounding variables. Poisson regression is commonly used in survival analysis and gives approximately the same parameter estimates and likelihood ratios as Cox proportional hazards regression when the length of follow-up is split into finer intervals (here we used 2 year intervals) [12, 13].

The details for the calculation of total alcohol intake have been described previously [14]. Briefly, the reported quantities of beer, wine, and spirits were converted to grams of alcohol using food composition data from the Swedish National Food Administration [14]. Total alcohol intake was categorized according to the distribution of the variable in the WLH population as categories (0, 0.1–5.0, 5.1–15, and >15 g/day), and as a continuous variable. The category of 0–5 g of alcohol per day corresponds to approximately 0 to 2 glasses of wine per week (1 glass = 1 dl; alcohol by volume = 10 %). Age was included as a categorical covariate.

We assessed the following potential confounding covariates: educational attainment (0–11 years, >11 years), history of breast cancer in mother and/or sister(s) (Yes/No), smoking habits (current, former, never smokers), physical activity at enrollment with five grade scale, age at menarche (years), menopausal status (Yes/No), time varying parity at baseline (0, 1, 2, 3, and >3), age at the first child birth (years), breast feeding duration (month), and oral contraceptive use (current/former/never). Age at the first birth was modeled using, for nulliparous women, zero and for parous women extracting the average age at the first birth from the age at the first birth. Self-reported BMI was categorized into two groups (<= 25 kg/m^2^ and >25 kg/m^2^). Age at menopause and the use of hormone replacement therapy (HRT) were both analyzed as time varying variables using the combined 1991 and 2003 follow-up data. Thus, women’s menopausal status and HRT use could change along the follow-up period. Participants were censored when death or immigration occurred after entry into the cohort, or at 31st December 2009, whichever came first. The RR of breast cancer comparing different groups of exposure and change in RR by level of modifying covariates was estimated together with associated two-sided 95 % confidence intervals.

We fitted models, for breast cancer overall as well as by ER/PR receptor status, and included parameters for the main effects and the interaction term between BMI (<= 25 kg/m2 and >25 kg/m2) and alcohol intake (0, 0–5, 5–15 and >15 g/day). We calculated RR for breast cancer comparing each combination of alcohol and BMI versus low BMI and an alcohol intake of 0 g/day. In the same model we tested the statistical hypothesis of no interaction using a likelihood ratio test.

All statistical tests were two-sided with α= 0.05. All models for overall breast cancer were repeated for ER+/PR+, ER+/PR-, and ER-/PR- breast cancers separately. We did not separately analyze the ER-/PR+ tumors owing to the small number.

## Results

Baseline characteristics of study participants are presented in Table 1. High alcohol intake was more common among women with high educational attainment, women who were former or current smokers, women with few children, and among those who had ever used oral contraceptives. The proportion of women with BMI > 25 kg/m^2^ was highest among non-drinking women. In addition, the alcohol intake was higher among women with BMI ≤25 kg/m^2^ (median = 2.6 g/day for BMI ≤25 kg/m^2^, median = 1.8 g/day for BMI > 25 kg/m^2^). During the follow-up period, a total of 1,385 breast cancer cases were ascertained. Among them, 718 tumors were ER+/ and PR +, 196 were ER+/PR-, 187 were ER -/PR- and 37 were ER-/PR+. Table 2 shows breast cancer risk by alcohol intake in the entire cohort as well as for different hormone receptor subtypes. Alcohol intake was not statistically significantly associated with breast cancer risk, either overall or in different hormone receptor subtypes. We did not find evidence of a non-linear relation between alcohol intake and breast cancer incidence and RR estimated by splines (data not shown).

**Table 1:** Baseline characteristics of the Swedish Women’s Lifestyle and Health study participants according to alcohol intake

	Alcohol intake (g/day)			
	0	0.1–5	5.1–15	>15
No. of participans	10,153	22,249	11,734	1,097
Age at enrollment (mean)	39.8	39.7	40.5	41.4
Educational attainment >11 years, (%)	46.3	56.3	61.4	61.7
History of breast cancer in mother and/or sister (%)	4.2	4.2	5.3	5.9
Smoking habits (%)				
Never	50.5	43.0	32.0	18.1
Former	28.2	35.3	40.7	40.7
Current	21.3	21.7	27.3	41.2
Age at menarche (mean)	12.9	13.0	13.0	13.1
Women with more than 3 children (%)	32.5	27.2	24.0	23.4
Age at first child birth among parous women (mean)	23.5	24.0	24.1	23.8
Total duration of breast feeding (months)	30.9	30.8	30.2	30.3
Ever oral contraceptive use (%)	75.1	85.0	88.2	89.7
Post-menopause (%)	4.4	3.8	4.3	3.7
Body mass index (>25 kg/m^2^, %)	31.9	24.1	21.1	24.6

**Table 2:** Alcohol intake and risk of breast cancer by hormone receptor status in the Swedish Women’s Lifestyle and Health study

Alcohol intake (g/day)	Total	0	0.1–5	5.1–15	>15	Per 5 g/day increase
No. of participants	45,233	10,153	22,249	11,734	1,097	
Person-year	758,430	151,813	354,589	221,531	30,497	
Total breast cancer (No.)	1,385	276	646	421	42	
RR (95 % CI)- unadjusted		1.0	1.07 (0.92–1.25)	1.24 (1.06–1.45)	1.29 (0.99–1.67)	1.02 (1.00–1.04)
RR (95 % CI)- adjusted		1.0	1.03 (0.89–1.20)	1.16 (0.99–1.36)	1.17 (0.90–1.53)	1.01 (0.98–1.04)
ER(+)/PR(+) breast cancer (No.)	718	140	353	205	19	
RR (95 % CI)- unadjusted		1.0	1.17 (0.95–1.46)	1.25 (1.00–1.56)	1.21 (0.83–1.77)	1.02 (0.98–1.05)
RR (95 % CI)- adjusted		1.0	1.13 (0.91–1.40)	1.17 (0.93–1.47)	1.11 (0.76–1.63)	1.01 (0.97–1.05)
ER(+)/PR(−) breast cancer (No.)	196	39	88	65	4	
RR (95 % CI)- unadjusted		1.0	0.99 (0.66–1.50)	1.31 (0.87–1.99)	1.04 (0.51–2.15)	1.01 (0.95–1.08)
RR (95 % CI)- adjusted		1.0	1.00 (0.66–1.51)	1.35 (0.89–2.06)	1.09 (0.53–2.25)	1.02 (0.95–1.08)
ER(−)/PR(−) breast cancer (No.)	187	45	83	51	8	
RR (95 % CI)- unadjusted		1.0	0.95 (0.64–1.42)	1.03 (0.67–1.57)	1.27 (0.64–2.52)	1.01 (0.92–1.10)
RR (95 % CI)- adjusted		1.0	0.90 (0.60–1.33)	0.91 (0.59–1.39)	1.04 (0.52–2.08)	0.98 (0.87–1.33)

Adjusted models were adjusted for educational attainment (0–11 years/>11 years), history of breast cancer in mother and/or sister (Yes/No), smoking habits (current/former/never smokers), age at menarche (years), parity at baseline (0, 1, 2, 3, and >3), age at the first child birth (years), total breast feeding duration (months), and oral contraceptive use (current/former/never)

*RR* relative risk, *CI* confidence interval, *ER* estrogen receptor, *PR* progesterone receptor

Overall breast cancer risk increased with increasing alcohol intake among women with BMI ≤25 kg/m^2^ (RR = 1.03, 95 % CI: 1.00–1.05 per 5 g/day increase), but not among women with BMI over 25 kg/m^2^. Similar patterns were observed for all subtypes of breast cancer, but statistical tests for interaction were significant only for ER-/PR- breast cancer (*p* = 0.04) (Table 3).

**Table 3:** Relative risks (RR) and their 95 % confidence intervals (CI) on the association between alcohol intake and breast cancer risk by body mass index (BMI) in the Swedish Women’s Lifestyle and Health study

					BMI
		<=25 kg/m^2^			>25 kg/m^2^
Alcohol intake (g/day)	No. of cases	RR (95 % CI)	No. of cases	RR (95 % CI)	p-interaction
Total breast cancer (*n* = 1,385)					
0	150	1.0	86	0.99 (0.76–1.29)	0.59
0.1–5	418	1.05 (0.87–1.26)	181	1.00 (0.80–1.24)	
5.1–15	339	1.19 (0.98–1.44)	135	1.08 (0.86–1.37)	
> 15	56	1.32 (0.97–1.81)	20	0.88 (0.55–1.41)	
Per 5 g/day increase		1.03 (1.00–1.05)		0.96 (0.88–1.04)	
ER(+)/PR(+) breast cancer (*n* = 718)					
0	71	1.0	46	1.08 (0.75–1.57)	0.69
0.1–5	217	1.14 (0.87–1.49)	109	1.23 (0.91–1.66)	
5.1–15	167	1.22 (0.92–1.61)	72	1.18 (0.85–1.65)	
> 15	26	1.28 (0.81–2.03)	10	0.90 (0.46–1.76)	
Per 5 g/day increase		1.03 (0.99–1.06)		0.93 (0.83–1.05)	
ER(+)/PR(−) breast cancer (*n* = 196)					
0	23	1.0	10	0.69 (0.33–1.45)	0.98
0.1–5	55	0.94 (0.58–1.53)	23	0.77 (0.43–1.39)	
5.1–15	55	1.29 (0.79–2.13)	20	0.98 (0.53–1.81)	
> 15	7	1.06 (0.45–2.50)	3	0.80 (0.24–2.69)	
Per 5 g/day increase		1.02 (0.96–1.09)		0.98 (0.82–1.18)	
ER(−)/PR(−) breast cancer (*n* = 187)					
0	17	1.0	19	1.95 (1.01–3.76)	0.04
0.1–5	60	1.29 (0.75–2.22)	21	1.01 (0.53–1.93)	
5.1-15	44	1.31 (0.74–2.30)	15	1.03 (0.51–2.08)	
> 15	10	1.96 (0.89–4.34)	1	0.36 (0.05–2.74)	
Per 5 g/day increase		1.02 (0.94–1.11)		0.80 (0.59–1.09)	

Adjusted models were adjusted for educational attainment (0–11 years/>11 years), history of breast cancer in mother and/or sister (Yes/No), smoking habits (current/former/never smokers), age at menarche (years), parity at baseline (0, 1, 2, 3, and >3), age at the first child birth (years), total breast feeding duration (months), and oral contraceptive use (current/former/never)

*ER* estrogen receptor, *PR* progesterone receptor

Additional analysis with menopausal status as time-varying variable or HRT use as time-varying variable did not change the results (data not shown).

## Discussion

After multivariable adjustment we did not observe a statistically significant risk of breast cancer in the combined group of lean and overweight women. However, we found that high intake of alcohol was associated with an increase in overall breast cancer risk only among women with BMI ≤25 kg/m^2^. It suggests that alcohol and obesity may not act as an additive manner on the breast cancer risk among pre-menopausal women. We also observed statistically significant effect modification of alcohol by body mass index on ER-/PR- breast cancer risk.

Three cohort studies reported similar results [15–17]. In both the EPIC study and the epidemiologic follow-up to the first National Health and Nutrition Examination Study (NHANES), the elevated risk by alcohol consumption was most prominent among both pre- and postmenopausal lean women (BMI <18.5 kg/m^2^ in EPIC and <22.5 kg/m^2^ in NHANES). In the California Teachers Study Cohort, normal weight or marginally overweight women (BMI < 27.3 kg/m^2^) whose alcohol consumption level was ≥ 20 g/day showed 40 % higher risk for breast cancer compared with nondrinking normal weight or marginally overweight women (95 % CI 1.10–1.80) [15]. The RR for nondrinking obese women was 1.21 (95 % CI 0.96–1.53), and that for obese women with ≥ 20 g/day alcohol consumption was 1.33 (95 % CI 0.87–2.04). Several case–control studies also suggested effect modification by body mass index, with the effect of moderate alcohol consumption limited to women with low or normal BMI [18, 19]. In a pooled-analysis, however, no significant effect modification by body mass index on the association between alcohol consumption and breast cancer risk was found [20, 21].

Alcohol and obesity probably share common biological mechanisms in breast carcinogenesis through circulating sex hormone levels. Both alcohol consumption and obesity measures (BMI and waist-hip ratio) were associated with circulating hormones, especially SHBG in a cross-sectional study [22]. The variation in SHBG was, however, larger by BMI groups (54.1 nmol/L for <20 kg/m^2^ vs. 31.7 nmol/L for >27.5 kg/m^2^) than by alcohol consumption level (51.1 nmol/L for non-drinker vs. 43.7 nmol/L for ≥ 16 g/day). We postulate that obese women whose SHBG level is already decreased may be less affected by alcohol intake.

Previous epidemiological studies reported associations between alcohol consumption and ER+ breast tumors, whereas weak or null associations were observed for ER- tumors [23]. The strongest association was observed for ER+/PR- tumors [23]. While the results were not statistically significant, the pattern of the point estimates in our study is similar to the previous meta-analysis. The mechanisms of stronger association between alcohol consumption and ER+/PR- tumors than ER+/PR+ are unknown. Although limited by low statistical power in the stratified analysis by hormonal receptor status, elevated breast cancer risk with higher alcohol consumption was observed for low BMI groups regardless of hormone receptor status of the tumor in our study. In addition, elevated risk with higher alcohol consumption among low BMI group and the highest relative risk among obese non-drinkers for ER-/PR- breast cancer suggested a potential interaction between alcohol intake and ER-/PR- tumors by BMI. This finding should be investigated further in other epidemiological studies. Ovarian hormones are essential for development of both hormone-dependent and hormone-independent breast tumors [24]. Controlled feeding studies suggest that alcohol intake increases circulating estrogen levels in both premenopausal [25–27] and postmenopausal women [28–31]. In contrast, moderate alcohol consumption is also linked to lower progesterone concentration in premenopausal women [26]. Proposed mechanisms of by which alcohol intake effects estrogen levels include an increased rate of aromatization of testosterone or a decreased rate of oxidation of estradiol to estrone [32]. However, only a few studies have reported alcohol consumption, body mass index and endogenous female hormone concentrations simultaneously in postmenopausal women [20, 33]. In our analysis, additional adjustment for exogenous hormone use did not change the association between alcohol consumption and breast cancer risk. Further study with comprehensive assessment of hormone levels and adiposity measures would help to explain the mechanisms of the excess risk of alcohol consumption in lean premenopausal women.

Strengths of our study include its prospective design, which minimizes potential for recall bias. Potential confounders of the association between alcohol consumption and breast cancer risk, such as family history of breast cancer, were adjusted for in the multivariate model. Breast cancer ascertainment by linkage to the registries ensures virtually complete follow-up. In addition, information on hormone receptor status was available for most of breast cancer patients with relatively high completeness. Another strength is the age structure of the cohort, since few cohort studies aimed to include predominantly premenopausal women [14].

Limitation of the study includes that information on alcohol consumption was assessed at baseline and follow-up surveys and may not accurately reflect long- term consumption habit. Analysis by hormone receptor status may lack statistical power due to small numbers of cases in each exposure groups.

## Conclusions

In conclusion, an increase in breast cancer risk with higher alcohol consumption was found for all breast cancers only among women with a BMI ≤25 kg/m^2^. The competing effects of obesity and alcohol consumption on circulating sex hormone levels may explain the results.

## Abbreviations

BMI: body mass index
EPIC: European Prospective Investigation into Cancer and Nutrition
ER: estrogen receptor
HRT: hormone replacement therapy
IARC: the International Agency for Research on Cancer
NHANES: National Health and Nutrition Examination Study
PR: progesterone receptor
RR: relative risk
SHBG: sex hormone binding globulin
WLH: Women’s Lifestyle and Health
